# Supplementary material for: Percolation transition of cooperative mutational effects in colorectal tumorigenesis
Source: Nat Commun. 2017 Nov 2;8:1270. doi: 10.1038/s41467-017-01171-6 (PMC5668266; doi:10.1038/s41467-017-01171-6)
Supplement: Supplementary file 1 — Supplementary Information [file 41467_2017_1171_MOESM1_ESM.pdf]

Supplementary Figure 1

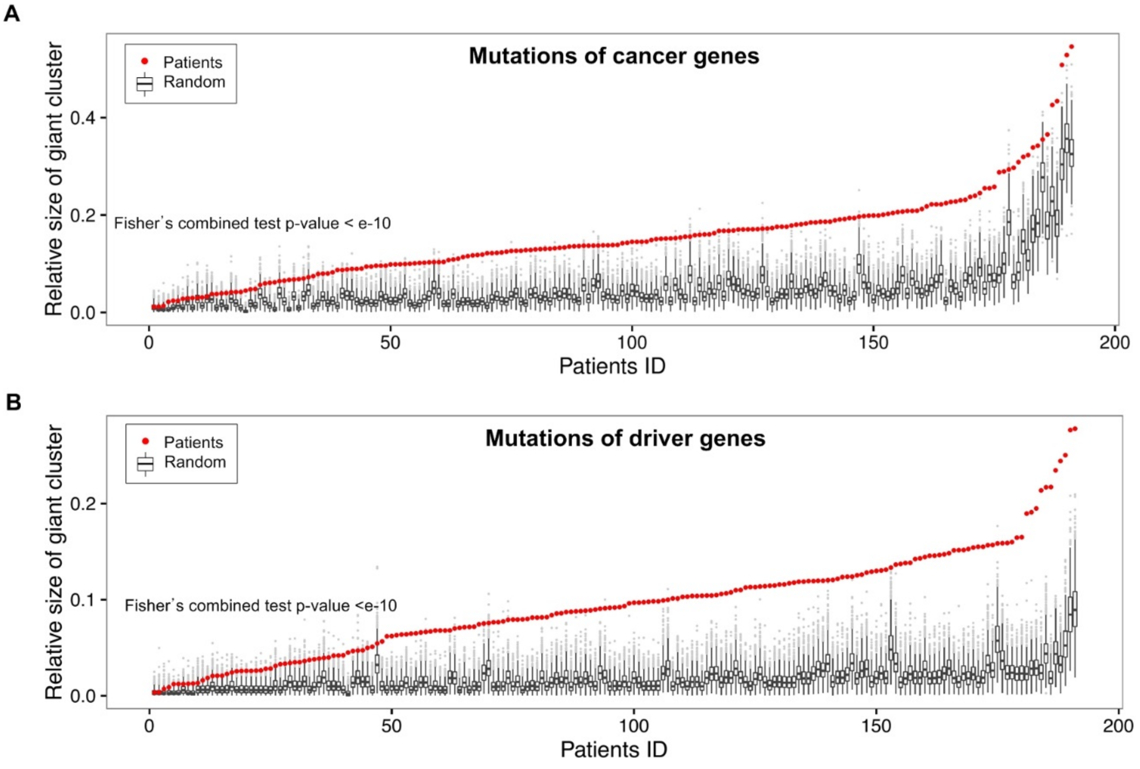

Supplementary Figure 1. The relative size of GC influenced by mutations of cancer genes (a) or driver genes (b) of patients compared to the random expectation (n = 1,000), where the same number of mutations for each patient was randomly selected.

Supplementary Figure 2

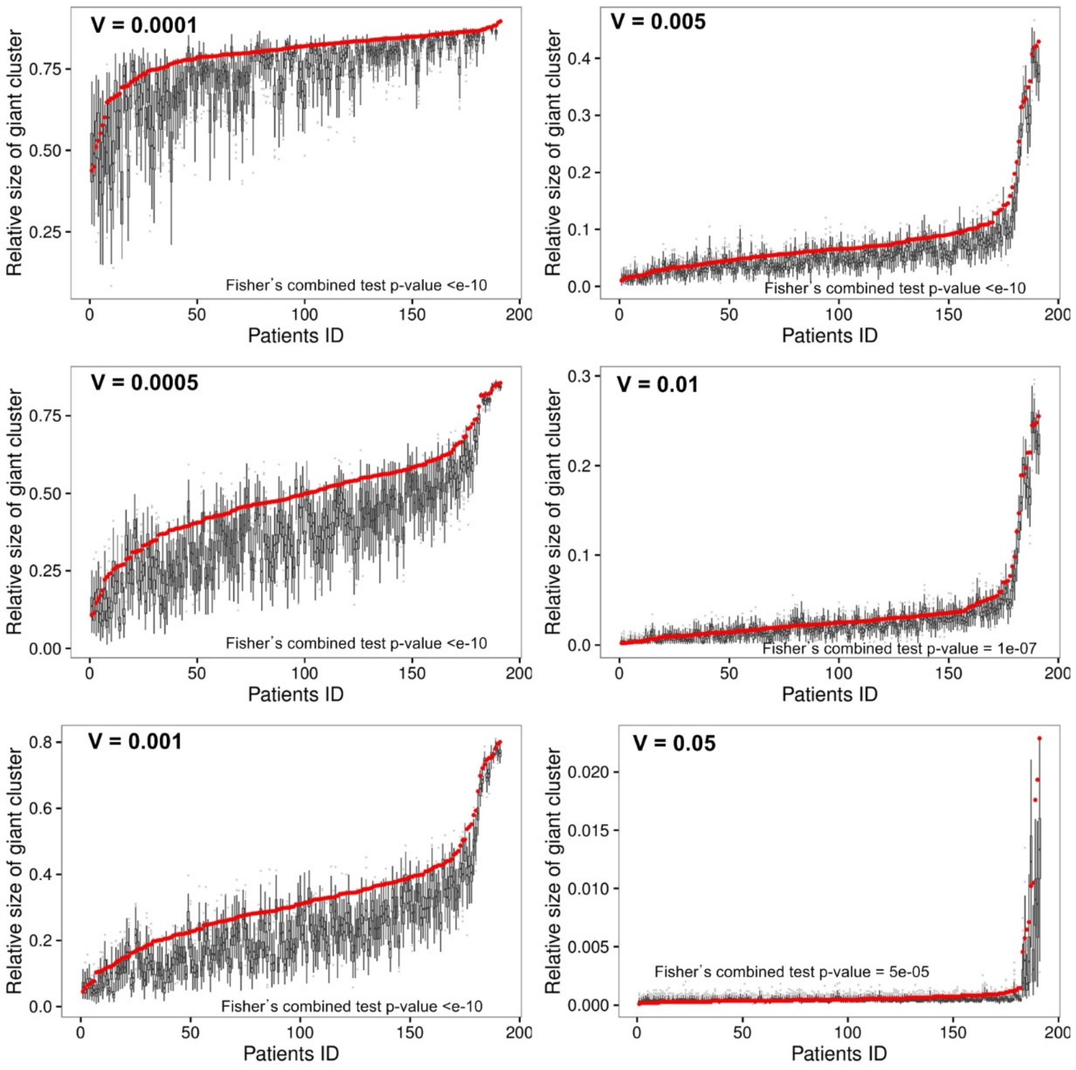

Supplementary Figure 2. The relative size of GC for patients compared to the random expectation (n = 1,000) for various thresholds of mutation influences.

Supplementary Figure 3

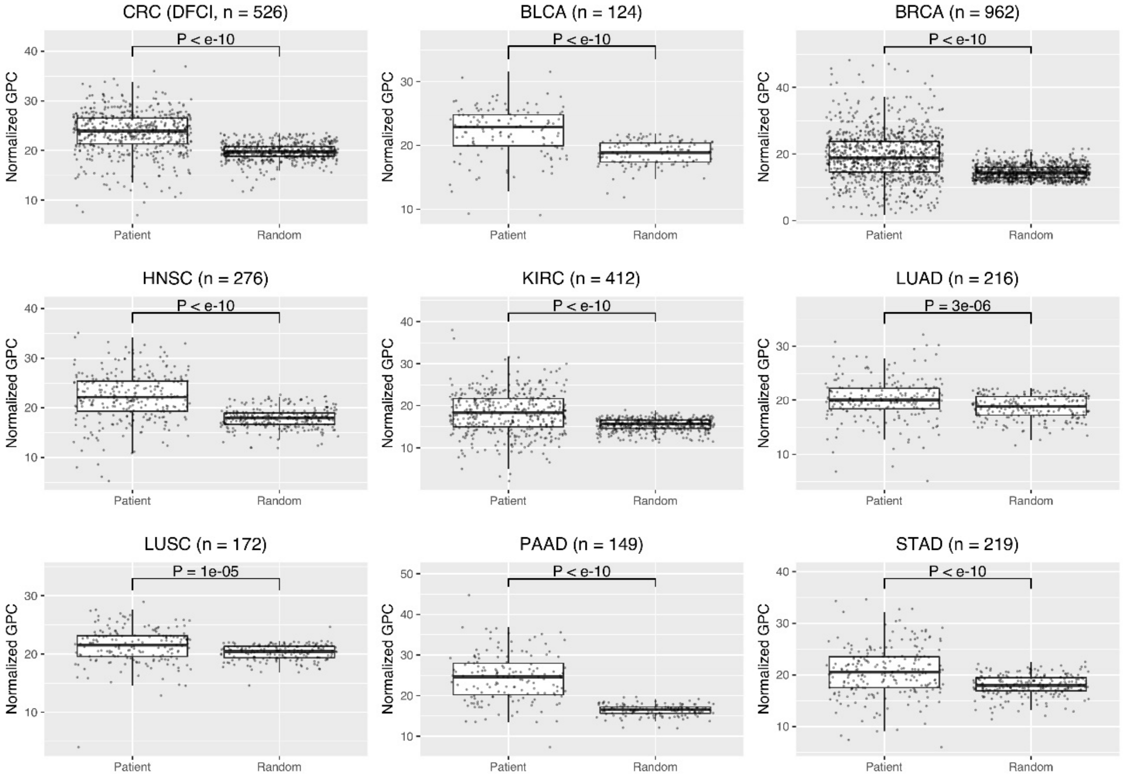

Supplementary Figure 3. The formation of a GPC in various solid tumors. The data that we considered include colorectal cancer (CRC from the DFCI dataset), urothelial bladder carcinoma (BLCA from the TCGA dataset), breast invasive carcinoma (BRCA from the TCGA dataset), head and neck squamous cell carcinoma (HNSC from the TCGA dataset), kidney renal clear cell carcinoma (KIRC from the TCGA dataset), lung adenocarcinoma (LUAD from the TCGA dataset), lung squamous cell carcinoma (LUSC from the TCGA dataset), pancreatic adenocarcinoma (PAAD from the TCGA dataset), and stomach adenocarcinoma (STAD from the TCGA dataset). The size of the GPC normalized by the number of mutations of each cancer patient was compared to the random expectation for which the same number of mutations for each patient was randomly selected, and the

averaged size of the normalized giant clusters (n = 100) was used. P-values were obtained with the t-test. The threshold of the mutation influence, V=0.005, was used.

Supplementary Figure 4

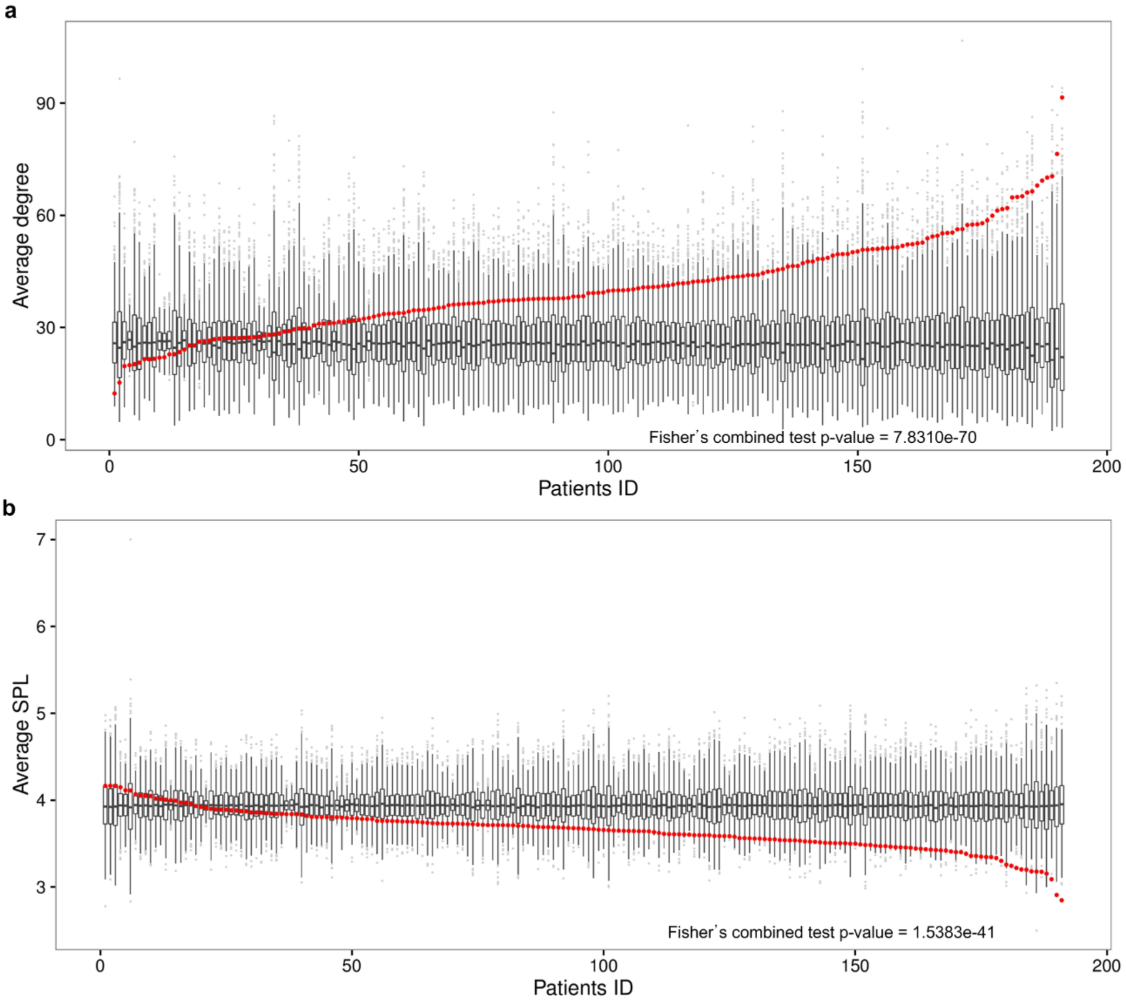

Supplementary Figure 4. The average degree of all the mutated genes (a) and the average shortest path length between all the mutation pairs (b) of each patient compared to the corresponding random expectation where the same number of mutations was randomly selected (n = 1,000).

Supplementary Figure 5

a

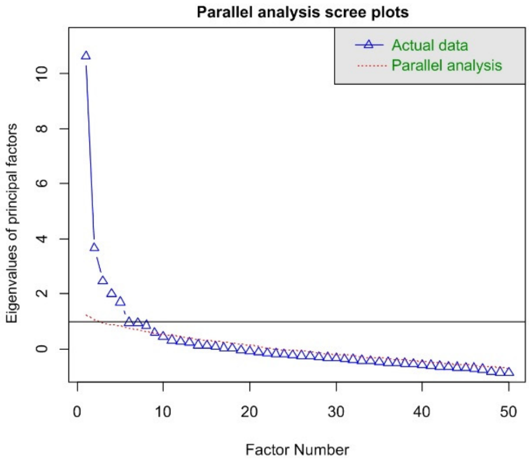

b

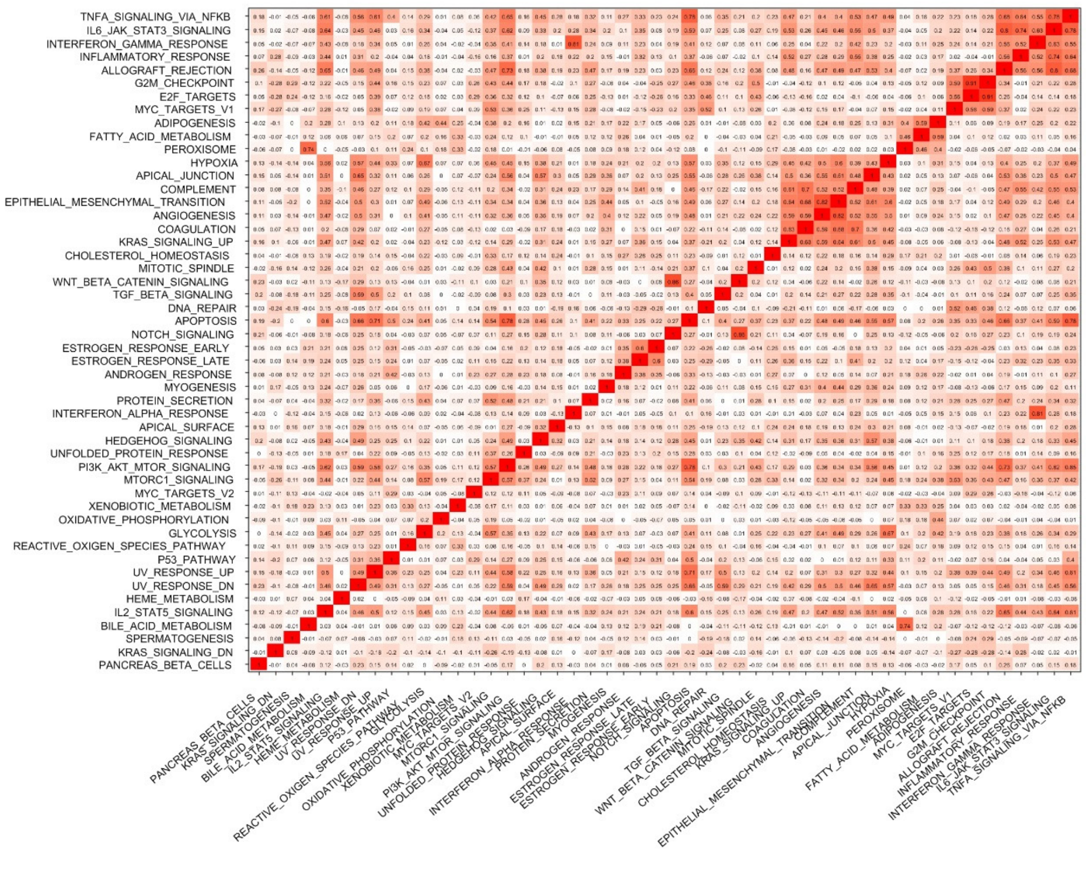

Supplementary Figure 5. Determining the number of factors in the factor analysis. (a) Parallel analysis scree plots. The factor analysis and parallel analysis were conducted on

50 different factor numbers. We determined the range of optimal factor numbers that satisfy both Kaiser's rule (i.e., all factor numbers should have eigenvalues >1) and the parallel analysis threshold (i.e., all factor numbers obtained from the parallel analysis should have greater eigenvalues than those from the factor analysis). Scree plot shows that maximum number of factors is five. (b) The correlation matrix of hallmark gene sets identifies several sets of correlated hallmark gene sets.

Supplementary Figure 6

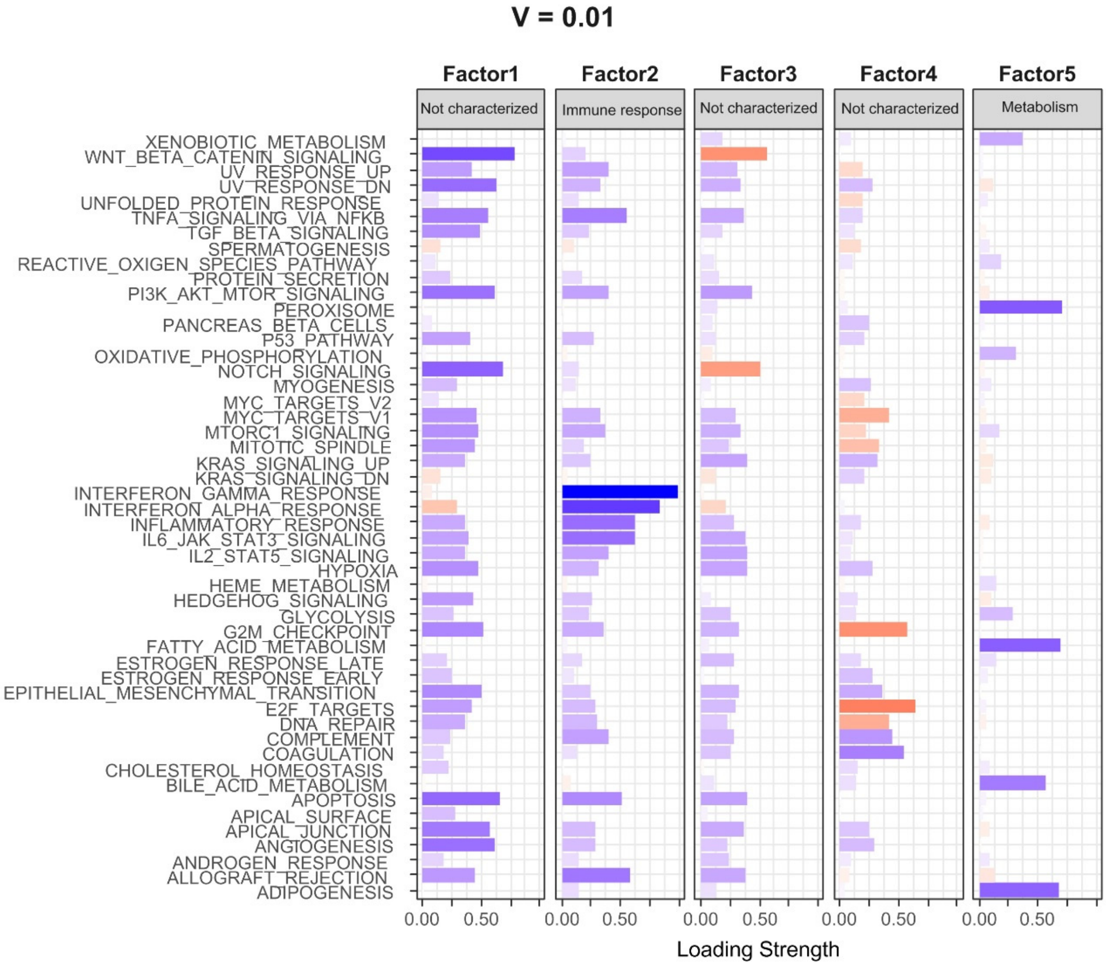

Supplementary Figure 6. Biological interpretation of identified factors for V = 0.01. Each bar indicates the loading strength of a hallmark gene set in each factor. Blue (red) bars represent positive (negative) values, and absolute values were used for negative values. Factor 2 and 5 were characterized as immune response and metabolism, respectively.

Supplementary Figure 7

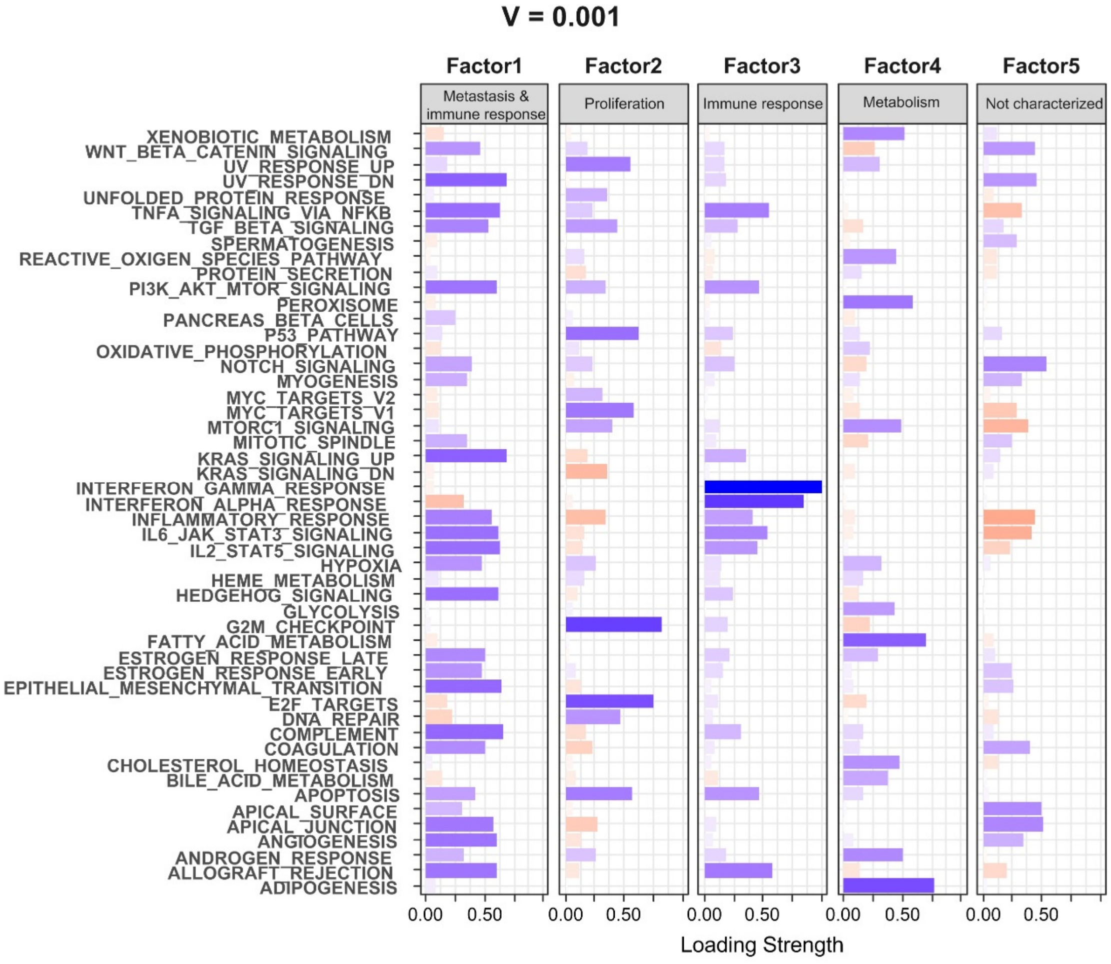

Supplementary Figure 7. Biological interpretation of identified factors for V = 0.001. Each bar indicates the loading strength of a hallmark gene set in each factor. Blue (red) bars represent positive (negative) values, and absolute values were used for negative values. Factor 1, 2, 3, and 4 were characterized as metastasis and immune response, proliferation, immune response, and metabolism, respectively.

Supplementary Figure 8

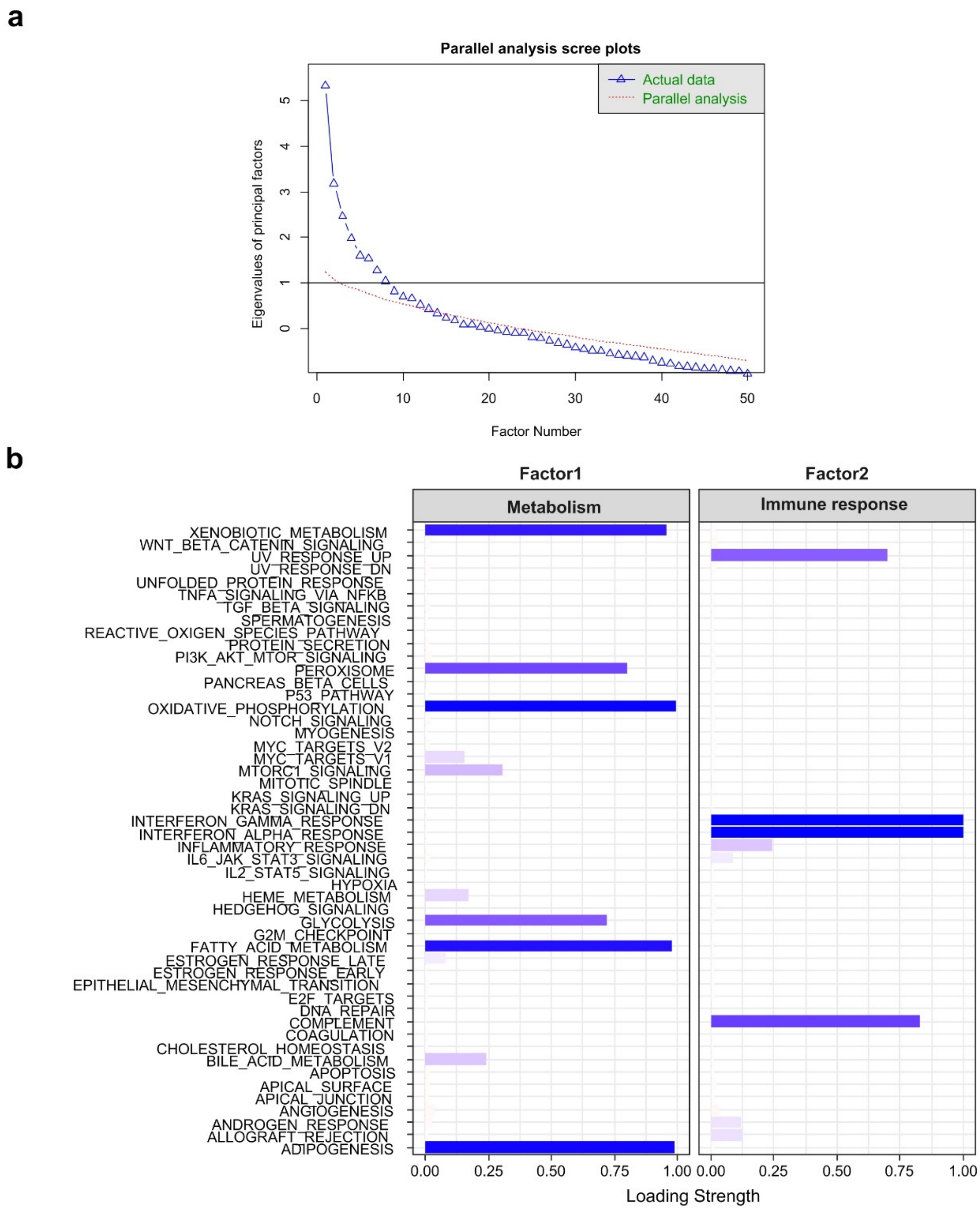

Supplementary Figure 8. The factor analysis for the propagation of cancer genes with a threshold V = 0.01. (a) Parallel analysis scree plots. The factor analysis and parallel analysis were conducted on 50 different factor numbers. Scree plot shows that maximum

number of factors is eight. (b) Biological interpretation of identified factors for the factor number of two. Each bar indicates the loading strength of a hallmark gene set in each factor. Blue (red) bars represent positive (negative) values, and absolute values were used for negative values. Factor 1 and 2 were characterized as metabolism and immune response, respectively.

Supplementary Figure 9

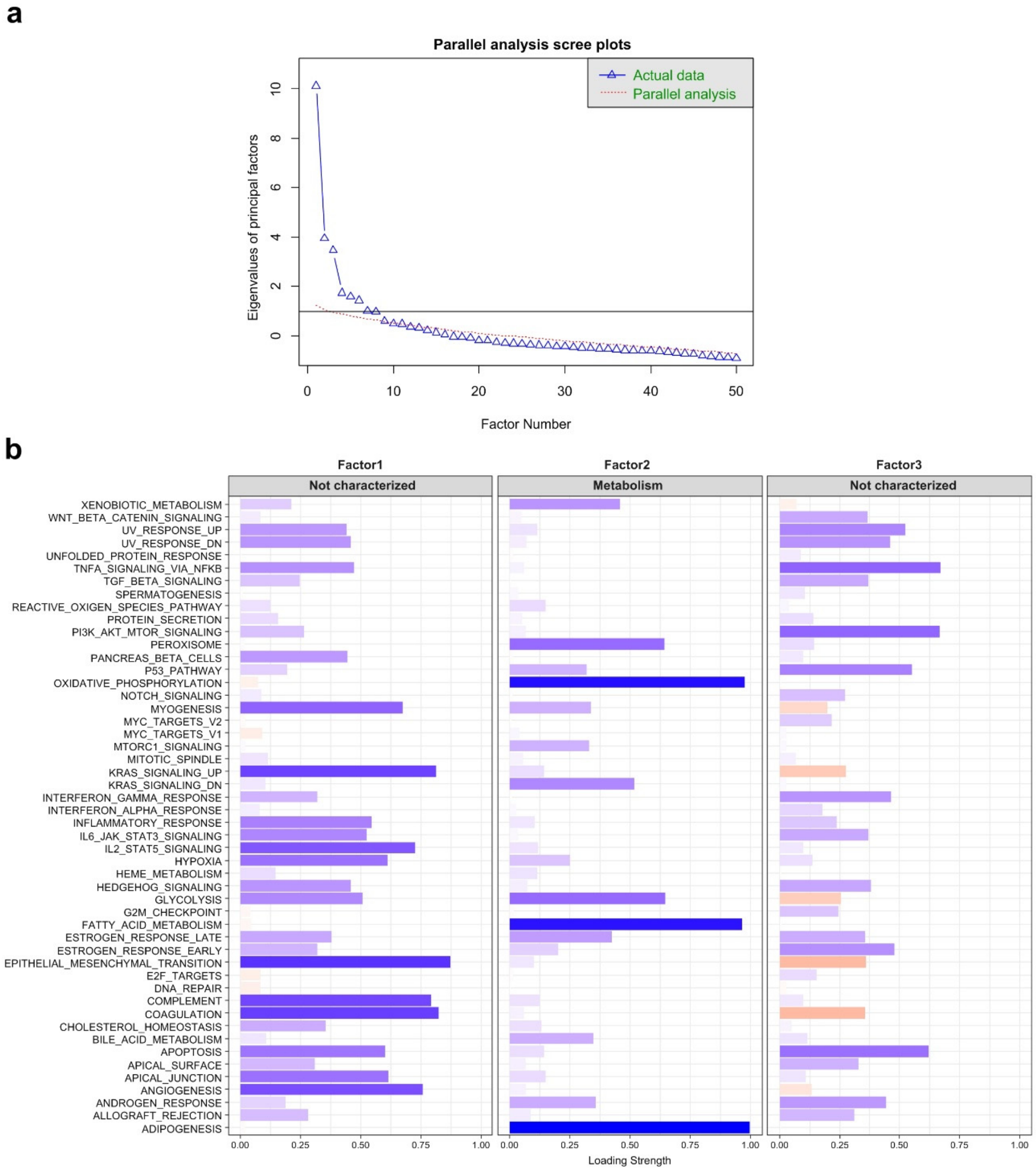

Supplementary Figure 9. The factor analysis for the propagation of cancer genes with a threshold V = 0.005. (a) Parallel analysis scree plots. The factor analysis and parallel analysis were conducted on 50 different factor numbers. Scree plot shows that maximum number of factors is six. (b) Biological interpretation of identified factors for the factor

number of three. Each bar indicates the loading strength of a hallmark gene set in each factor. Blue (red) bars represent positive (negative) values, and absolute values were used for negative values. Factor 2 were characterized as metabolism.

Supplementary Figure 10

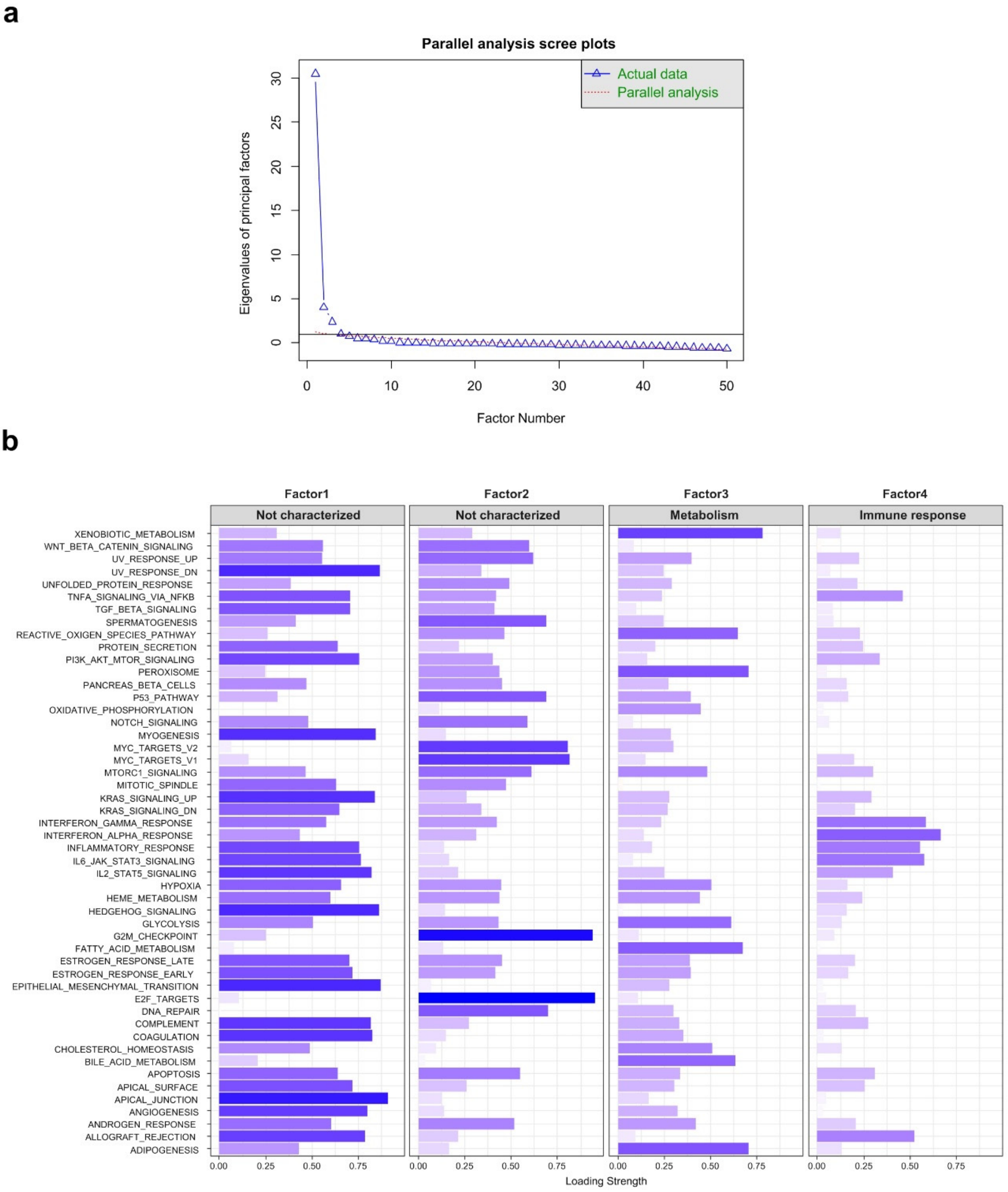

Supplementary Figure 10. The factor analysis for the propagation of cancer genes with a threshold V = 0.001. (a) Parallel analysis scree plots. The factor analysis and parallel analysis were conducted on 50 different factor numbers. Scree plot shows that maximum number of factors is four. (b) Biological interpretation of identified factors for the factor

number of four. Each bar indicates the loading strength of a hallmark gene set in each factor. Blue (red) bars represent positive (negative) values, and absolute values were used for negative values. Factor 3 and 4 were characterized as metabolism and immune response, respectively.

Supplementary Figure 11

a

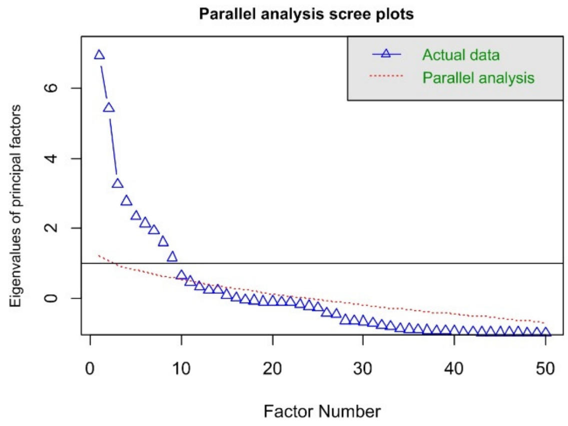

b

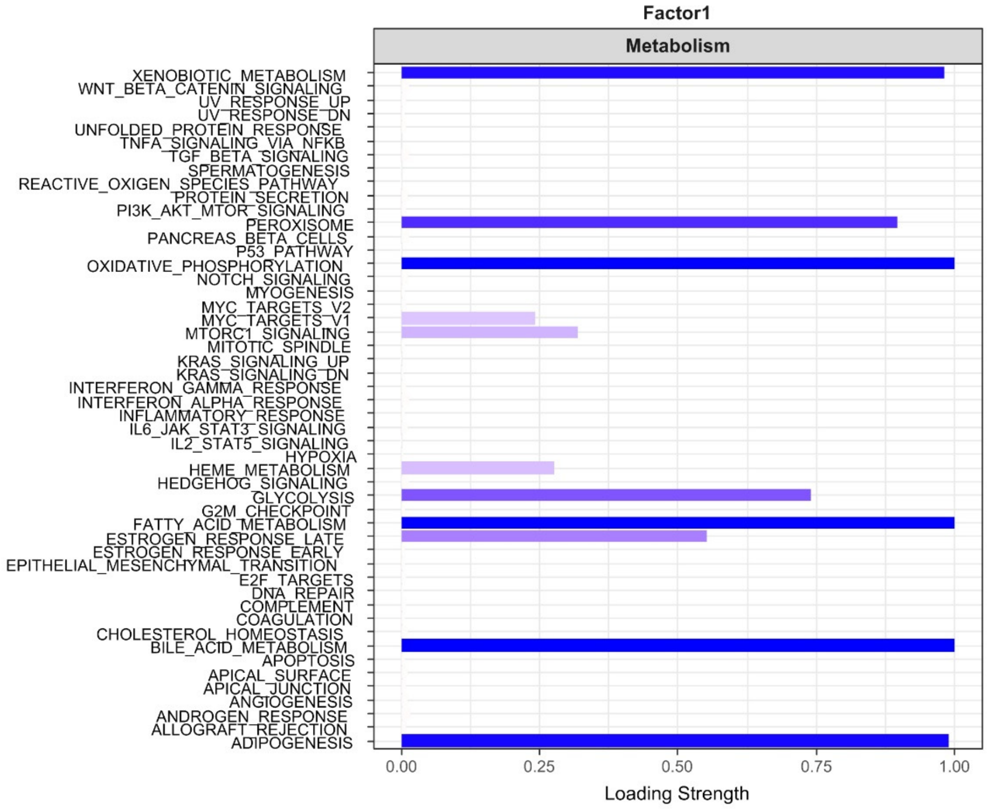

Supplementary Figure 11. The factor analysis for the propagation of driver genes with a threshold V = 0.01. (a) Parallel analysis scree plots. The factor analysis and parallel analysis were conducted on 50 different factor numbers. Scree plot shows that maximum number of factors is nine. (b) Biological interpretation of identified factors for the factor

number of one. Each bar indicates the loading strength of a hallmark gene set in each factor. Blue (red) bars represent positive (negative) values, and absolute values were used for negative values. Factor 1 was characterized as metabolism.

Supplementary Figure 12

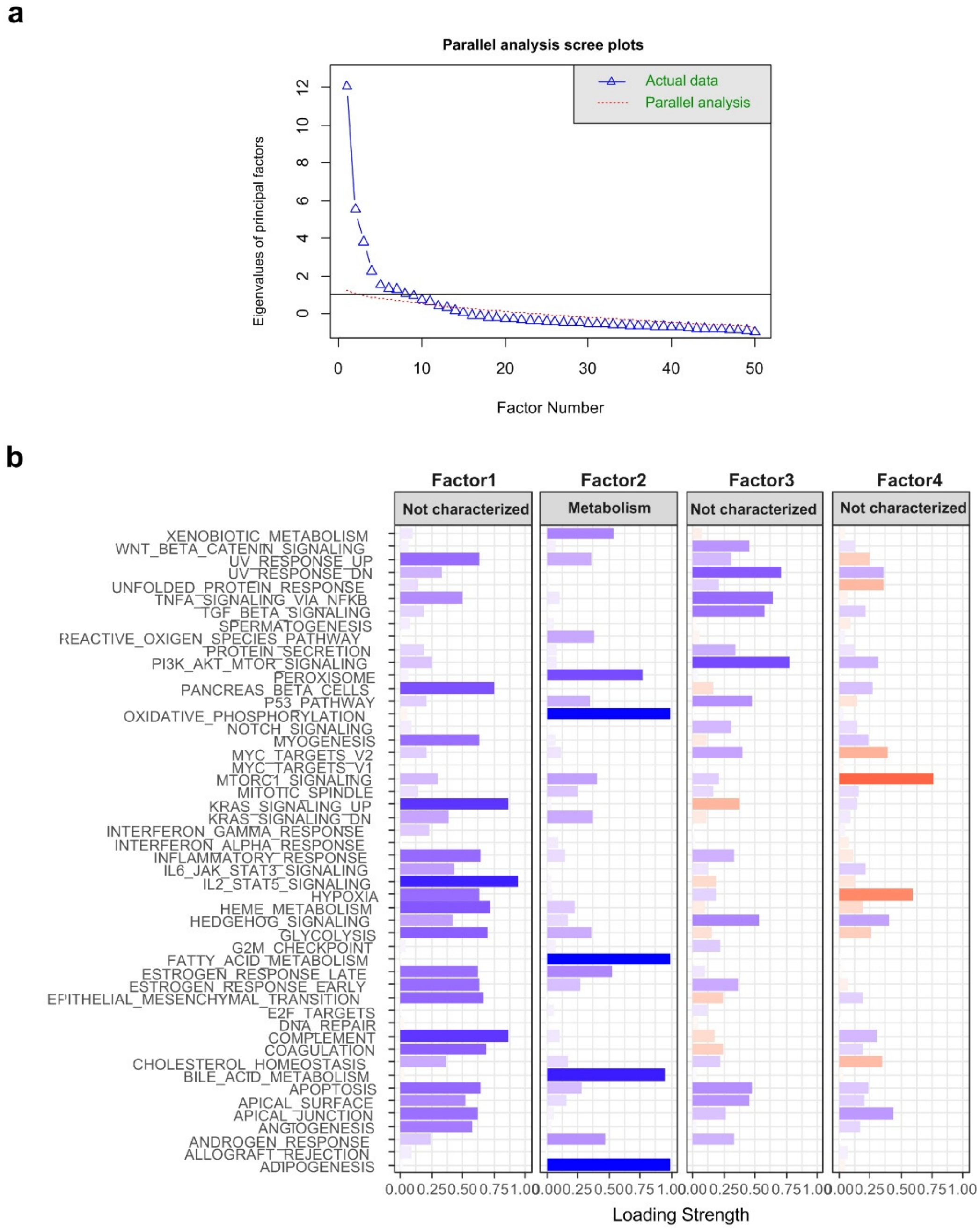

Supplementary Figure 12. The factor analysis for the propagation of driver genes with a threshold V = 0.005. (a) Parallel analysis scree plots. The factor analysis and parallel analysis were conducted on 50 different factor numbers. Scree plot shows that maximum number of factors is eight. (b) Biological interpretation of identified factors for the factor

number of four. Each bar indicates the loading strength of a hallmark gene set in each factor. Blue (red) bars represent positive (negative) values, and absolute values were used for negative values. Factor 2 was characterized as metabolism.

Supplementary Figure 13

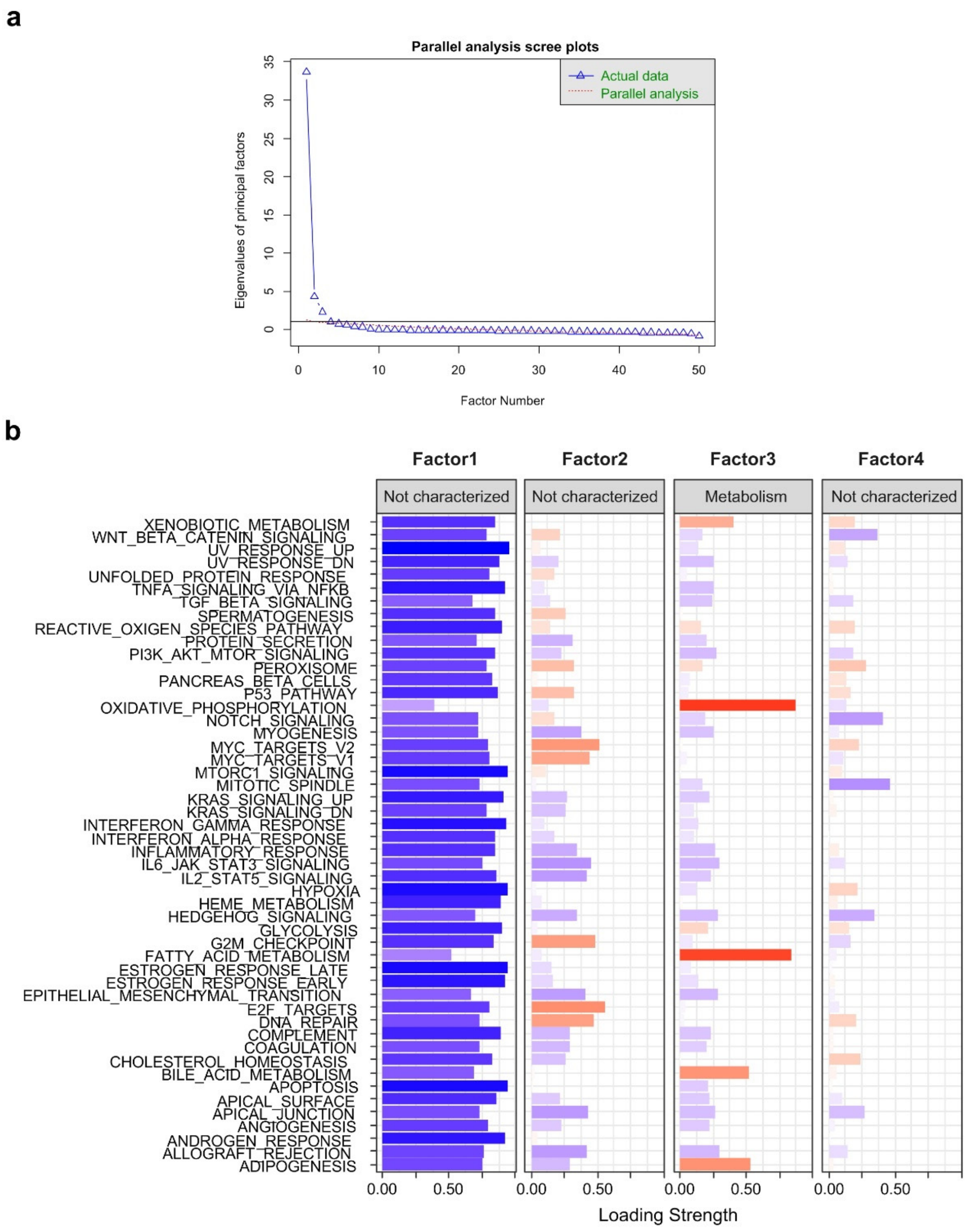

Supplementary Figure 13. The factor analysis for the propagation of driver genes with a threshold V = 0.001. (a) Parallel analysis scree plots. The factor analysis and parallel analysis were conducted on 50 different factor numbers. Scree plot shows that maximum number of factors is four. (b) Biological interpretation of identified factors for the factor

number of four. Each bar indicates the loading strength of a hallmark gene set in each factor. Blue (red) bars represent positive (negative) values, and absolute values were used for negative values. Factor 3 was characterized as metabolism.

Supplementary Figure 14

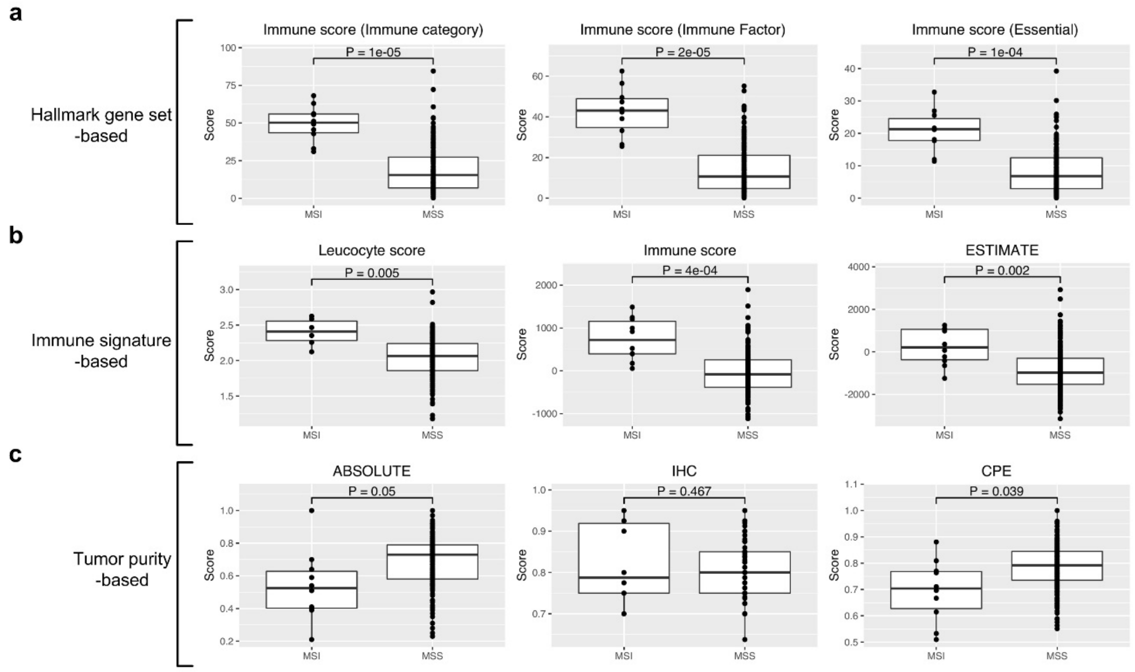

Supplementary Figure 14. Comparison of immune scores between the MSI and MSS groups. Three types of immune scores were used based on the Hallmark gene set (a), immune signature (b), and tumor purity (c). P-values were obtained with the t-test.

Supplementary Figure 15

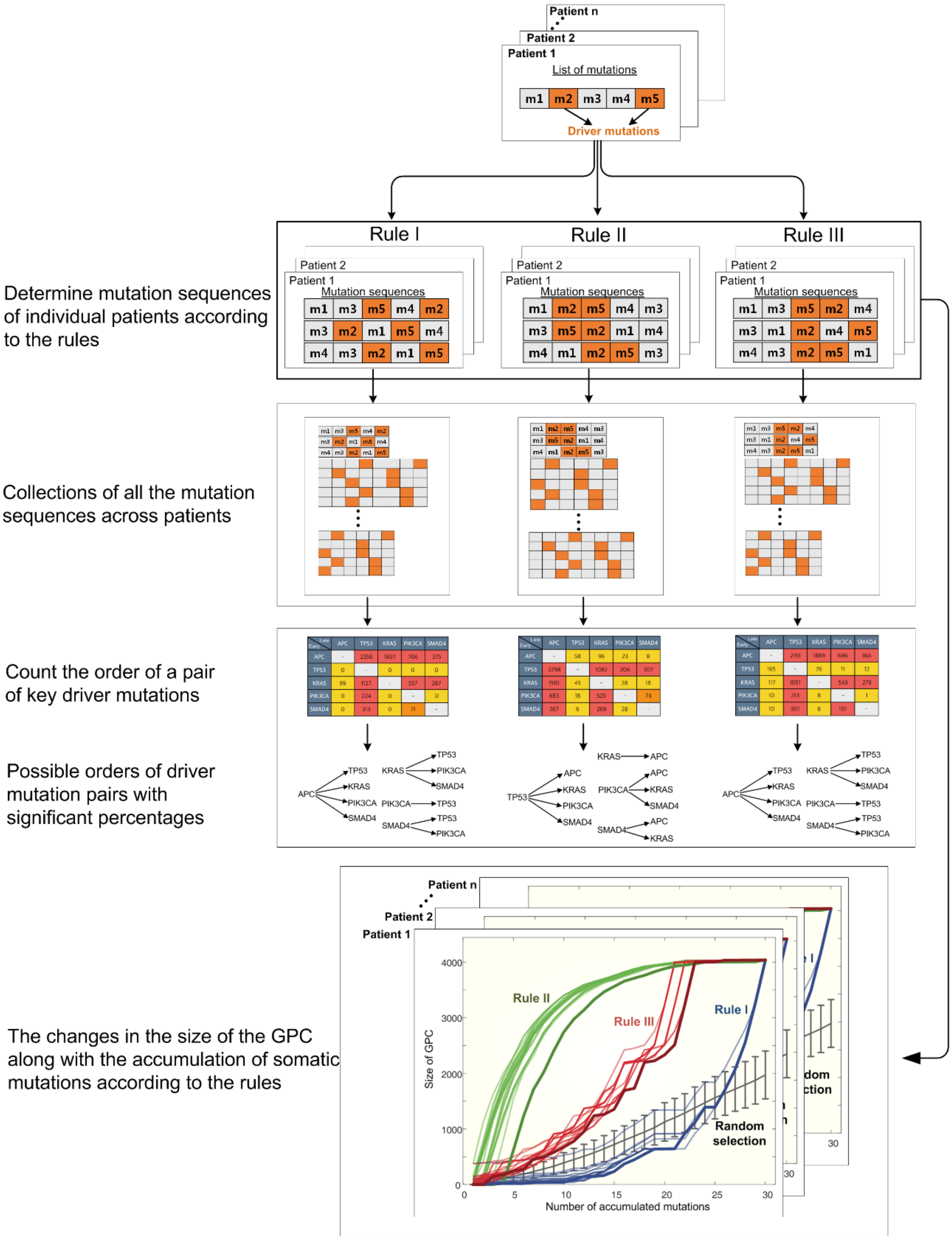

Supplementary Figure 15. Flowchart of the identification of mutation sequences and the simulation of the GPC according to the rules. From the mutation profiles of individual patients, we determined mutation sequences of each patient according to the three rules. All the mutations except driver mutations were selected as an initial mutation. We collected all the mutation sequences across patients and then counted the order of a pair of key driver mutations. Possible orders of driver mutation pairs with significant percentages were displayed. For each mutation sequence of patients, we calculated the size of the GPC along with the accumulation of somatic mutations.

Supplementary Figure 16

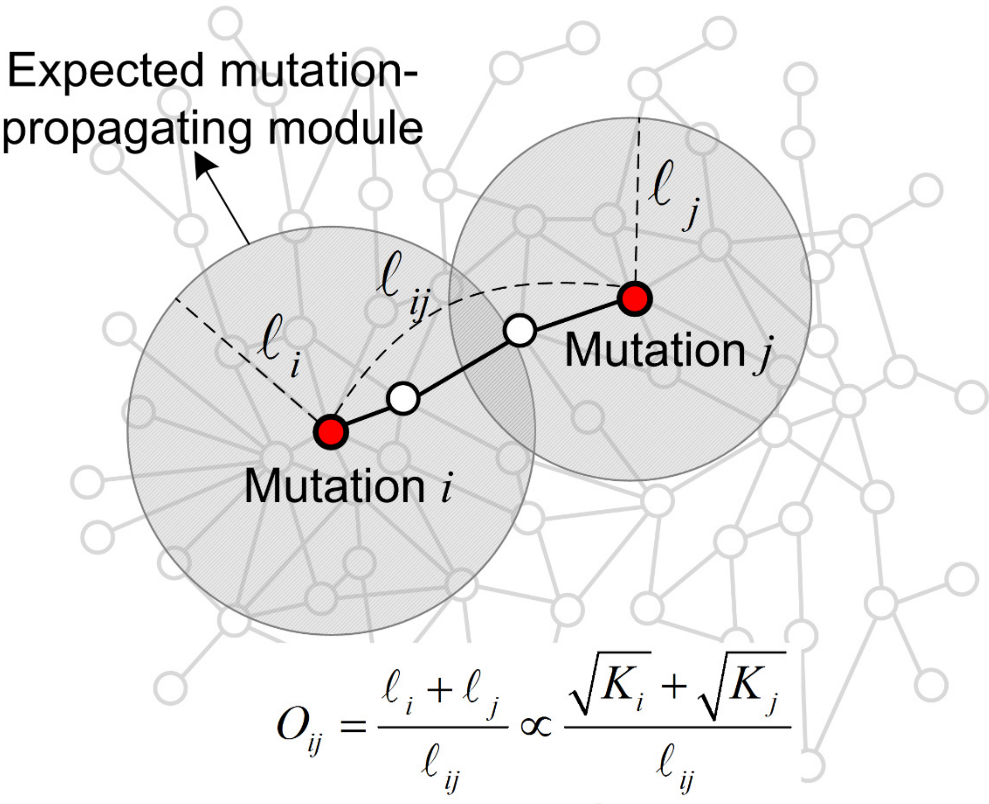

Supplementary Figure 16. Schematic of the degree of overlap between a pair of mutations. The degree of overlap between a pair of genes is determined only by the topological properties of them without applying the network propagation, i.e., the shortest path length between them and their node degrees. The overlap index based on the network propagation, such as the Jaccard index in Fig. 2c, cannot measure the degree of overlap when two mutation-propagating modules are not overlapped, whereas the overlap index based on the network topology can measure the probability of two mutations to be overlapped even when two mutations are located extremely far from each other. When a pair of mutations, i and j, are selected, the degree of overlap can be defined as $O_{ij} = (\ell_i + \ell_j) / \ell_{ij}$, where ℓ_i is the radius of an expected mutation-propagating module

of mutation i and ℓ_{ij} is the shortest path length between i and j. If we assume that the spatial distribution of nodes in the network is uniform, the size of mutation-propagating module of i would be proportional to the area of expected circle of the module, $S(i) \sim \pi \ell_i^2$. If we select the appropriate threshold for mutation influence such that the size of each module is proportional to its degree, $S(i) \sim aK_i$, where K_i is the node degree of i and a is a constant, then we would obtain $\ell_i \sim \sqrt{K_i}$, therefore consequently leading to the relation, the degree of overlap $O_{ij} \propto (\sqrt{K_i} + \sqrt{K_j}) / \ell_{ij}$.

Supplementary Figure 17

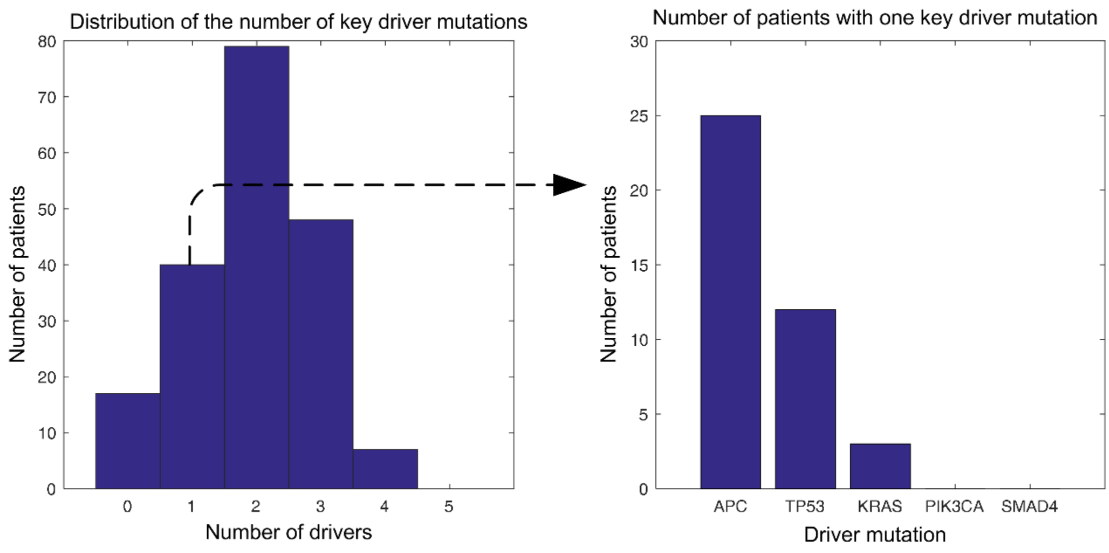

Supplementary Figure 17. Distribution of the number of key driver mutations in each patient. Key driver mutations include the most commonly observed mutations in colorectal cancer such as AKT, TP53, PIK3CA, KRAS, and SMAD4. Right figure shows the number of patients that have the corresponding driver mutation among 40 patients having a single key driver mutation. These, therefore, indicate that the probability for the first mutation occurring in APC might be high.

Supplementary Figure 18

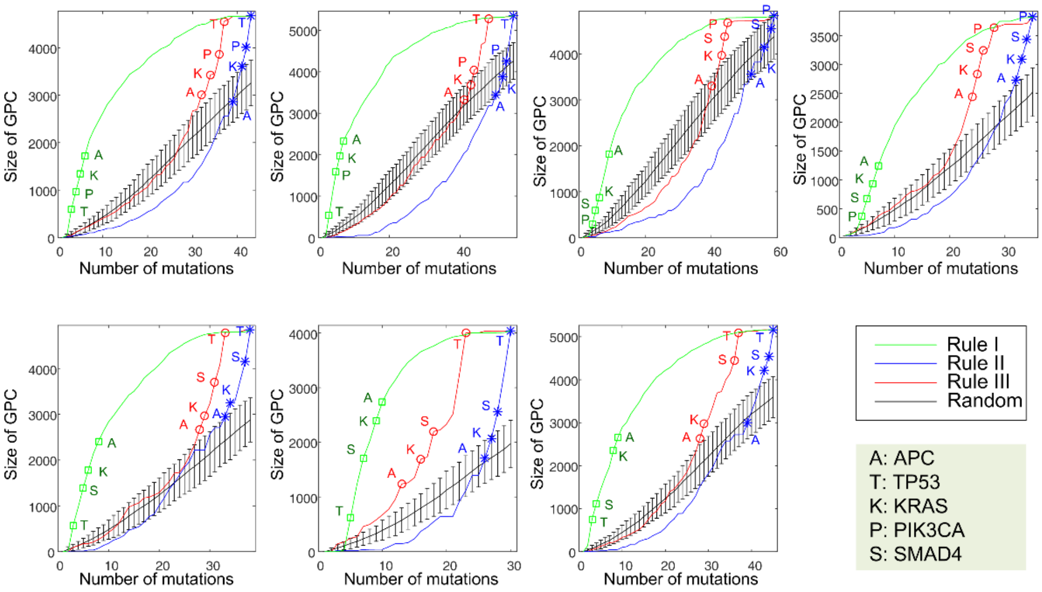

Supplementary Figure 18. The changes in the size of the GPC along with the accumulation of somatic mutations according to the rules for 7 patients who have 4 key driver mutations. For comparison of the rules and the random expectation, we generated 100 mutation sequences among randomly selected genes. Driver mutations are denoted at the corresponding order of occurrence of mutations in each rule.

Supplementary Figure 19

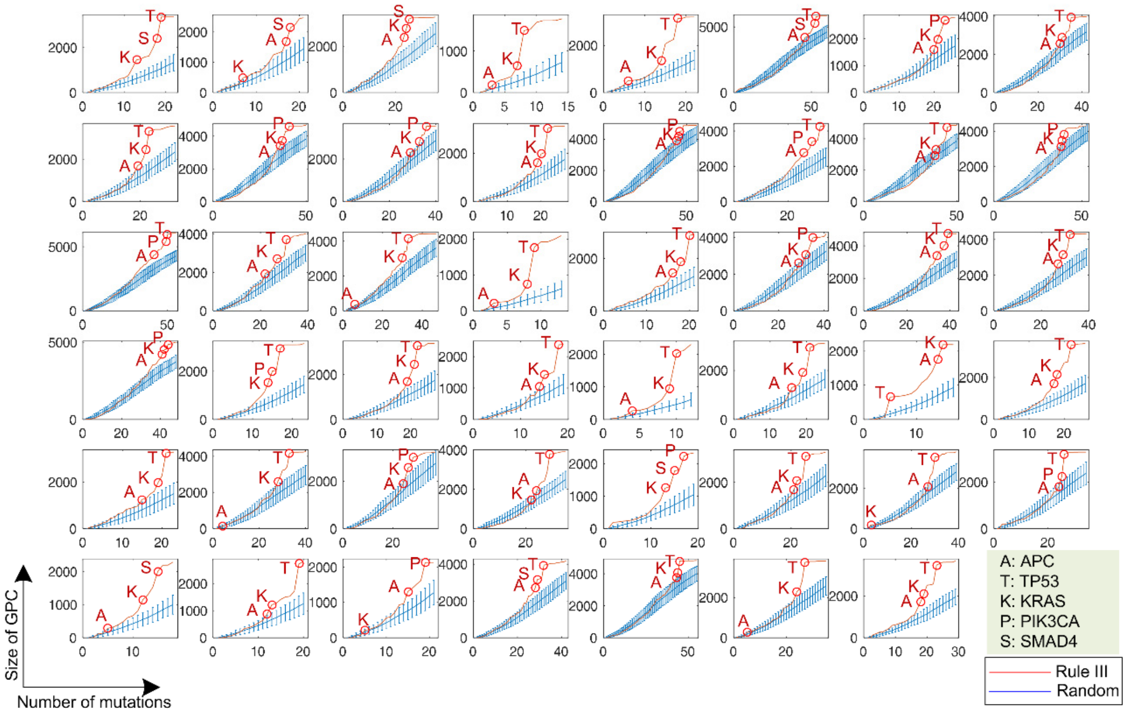

Supplementary Figure 19. The changes in the size of the GPC along with the accumulation of somatic mutations according to the third rule for 47 patients who have 3 key driver mutations. For comparison of the rule and the random expectation, we generated 100 mutation sequences among randomly selected genes. Driver mutations are denoted by circles at the corresponding order of occurrence of mutations in each rule.

Supplementary Figure 20

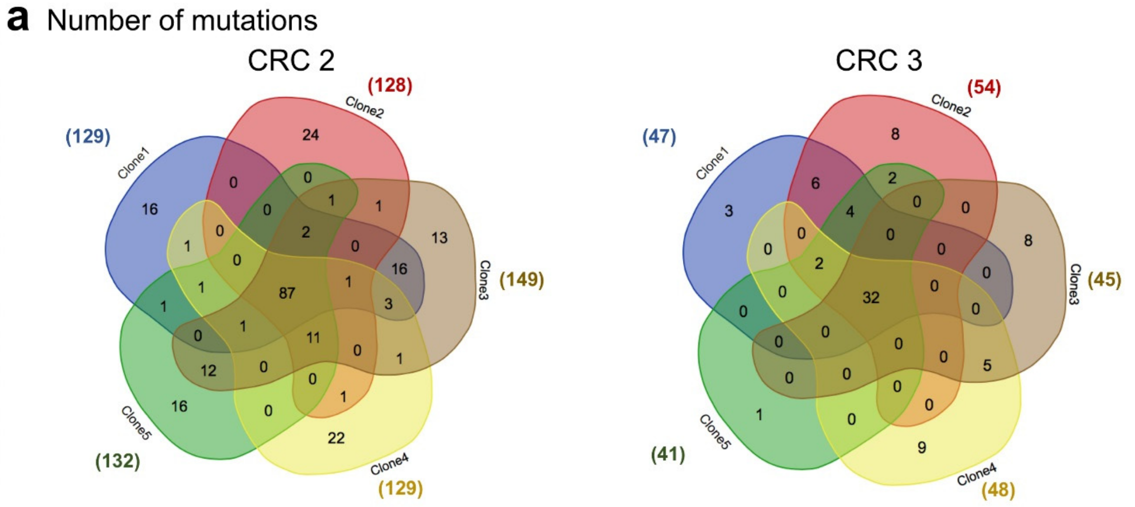

CRC2	Number of mutations	Cancer genes
Common	87 (37%)	5 (45%)
Private	144 (63%)	6 (55%)

CRC3	Number of mutations	Cancer genes
Common	32 (40%)	3 (75%)
Private	48 (60%)	1 (25%)

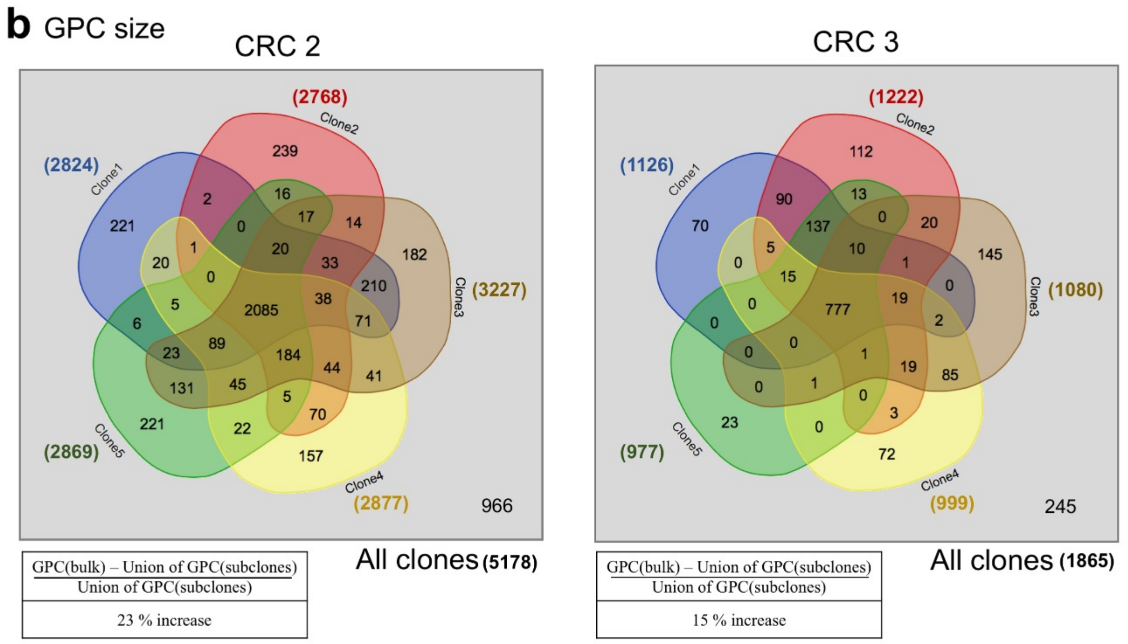

GPC(bulk) – Union of GPC(subclones) / Union of GPC(subclones)
23 % increase

GPC(bulk) – Union of GPC(subclones) / Union of GPC(subclones)
15 % increase

Supplementary Figure 20. Influence of tumor heterogeneity on the formation of GPC. (a) The Venn diagram shows the distribution of the mutation profiles of five subclones in the CRC2 (left) and CRC3 (right) samples. Each number represents the number of mutations that some clones share. (b) The Venn diagram shows the distribution of the gene list

contained in the GPC of five subclones in the CRC2 (left) and CRC3 (right) samples. Each number represents the number of genes that some clones share in their GPC. The square box indicates the GPC of the bulk which includes all the mutations of the subclones. The average expression profile of TCGA colorectal cancer patients was used to extract the colon cancer specific average PPI network.

Supplementary Figure 21

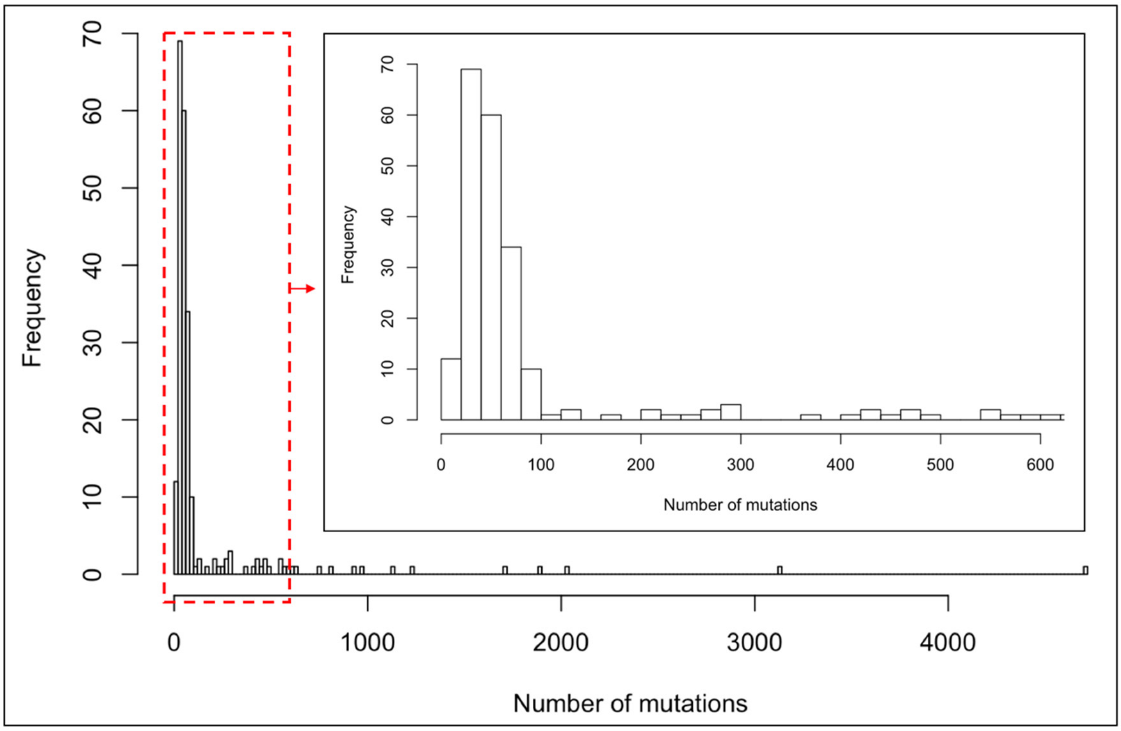

Supplementary Figure 21. Distribution of the number of mutations for 223 cancer patients. The inlet shows the expanded range where the number of mutations is relatively small (red dashed box).

Supplementary Figure 22

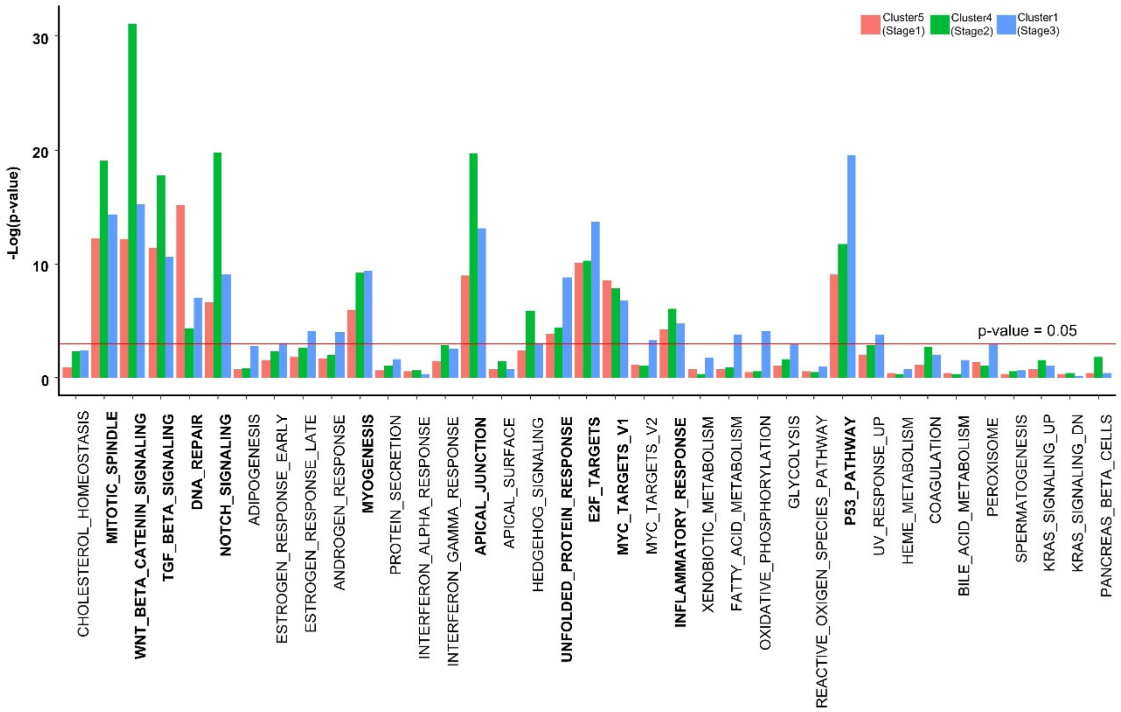

Supplementary Figure 22. The selected hallmark gene sets that are statistically significant in all three clusters. By applying the mRMR method, 37 hallmark gene sets were first selected, and then 12 hallmark gene sets that are statistically significant in cluster 5, 4, and 1 were selected to find out the phenotypic characteristics of each cluster.

Supplementary Table 1. Summary of the hallmark gene sets

No.	Hallmark gene sets	No.	Hallmark gene sets
1	TNFA_SIGNALING_VIA_NFKB	26	MTORC1_SIGNALING
2	HYPOXIA	27	E2F_TARGETS
3	CHOLESTEROL_HOMEOSTASIS	28	MYC_TARGETS_V1
4	MITOTIC_SPINDLE	29	MYC_TARGETS_V2
5	WNT_BETA_CATENIN_SIGNALING	30	EPITHELIAL_MESENCHYMAL_TRANSITION
6	TGF_BETA_SIGNALING	31	INFLAMMATORY_RESPONSE
7	IL6_JAK_STAT3_SIGNALING	32	XENOBIOTIC_METABOLISM
8	DNA_REPAIR	33	FATTY_ACID_METABOLISM
9	G2M_CHECKPOINT	34	OXIDATIVE_PHOSPHORYLATION
10	APOPTOSIS	35	GLYCOLYSIS
11	NOTCH_SIGNALING	36	REACTIVE_OXIGEN_SPECIES_PATHWAY
12	ADIPOGENESIS	37	P53_PATHWAY
13	ESTROGEN_RESPONSE_EARLY	38	UV_RESPONSE_UP
14	ESTROGEN_RESPONSE_LATE	39	UV_RESPONSE_DN
15	ANDROGEN_RESPONSE	40	ANGIOGENESIS
16	MYOGENESIS	41	HEME_METABOLISM
17	PROTEIN_SECRETION	42	COAGULATION
18	INTERFERON_ALPHA_RESPONSE	43	IL2_STAT5_SIGNALING
19	INTERFERON_GAMMA_RESPONSE	44	BILE_ACID_METABOLISM
20	APICAL_JUNCTION	45	PEROXISOME
21	APICAL_SURFACE	46	ALLOGRAFT_REJECTION
22	HEDGEHOG_SIGNALING	47	SPERMATOGENESIS
23	COMPLEMENT	48	KRAS_SIGNALING_UP
24	UNFOLDED_PROTEIN_RESPONSE	49	KRAS_SIGNALING_DN
25	PI3K_AKT_MTOR_SIGNALING	50	PANCREAS_BETA_CELLS
