# Supplementary material for: Percolation transition of cooperative mutational effects in colorectal tumorigenesis
Source: Nat Commun. 2017 Nov 2;8:1270. doi: 10.1038/s41467-017-01171-6 (PMC5668266; doi:10.1038/s41467-017-01171-6)
Supplement: Supplementary file 3 — Description of Additional Supplementary Files [file 41467_2017_1171_MOESM3_ESM.pdf]

Description of Additional Supplementary Files

File Name: Supplementary Data 1
Description: **Hallmark gene sets of each factor for different predefined factor numbers.**

File Name: Supplementary Data 2
Description: **Patient information including tumor stages, CMS, and MSI status.** We obtained tumor stage and MSI information from the cBioPortal website and CMS information from the Colorectal Cancer Subtype Consortium (CRCSC) website (https://www.synapse.org/#!Synapse:syn4978511.1). NOLBL means that the patient is not classified as any CMS type.
